# Clinical effects and correlates of standard rTMS and theta burst stimulation (TBS) on suicidal ideation in late-life depression

**DOI:** 10.1192/j.eurpsy.2025.10049

**Published:** 2025-06-27

**Authors:** Hyewon H. Lee, Katharina Göke, Rafae A. Wathra, Benoit Mulsant, Alisson P. Trevizol, Jonathan Downar, Shawn M. McClintock, Sean M. Nestor, Yoshihiro Noda, Tarek K. Rajji, Zafiris J. Daskalakis, Daniel M. Blumberger

**Affiliations:** 1 Temerty for Therapeutic Brain Intervention and Campbell Family Research Institute, Centre for Addiction and Mental Health, Toronto, ON, Canada; 2 Department of Psychiatry, Temerty Faculty of Medicine, University of Toronto, Toronto, ON, Canada; 3 Institute of Medical Science, University of Toronto, Toronto, ON, Canada; 4 Department of Psychiatry, Baycrest Health Sciences, Toronto, ON, Canada; 5 Department of Psychiatry, University of Texas Southwestern Medical Center, Dallas, TX, USA; 6 Harquail Centre for Neuromodulation, Sunnybrook Health Sciences Center, Toronto, ON, Canada; 7 Department of Neuropsychiatry, Faculty of Medicine, Keio University School of Medicine, Tokyo, Japan; 8 Department of Psychiatry, University of California, San Diego Health, San Diego, CA, USA

**Keywords:** depression, late life, rTMS, suicidal ideation, theta burst stimulation

## Abstract

**Background:**

Previous studies have shown that repetitive transcranial magnetic stimulation (rTMS) can treat suicidal symptoms; however, the effects of rTMS on suicidal ideation (SI) in late-life depression (LLD) have not been well-characterized, particularly with theta burst stimulation (TBS).

**Methods:**

Data were analyzed from 84 older adults with depression from the FOUR-D trial (ClinicalTrials.gov identifier: NCT02998580), who received either bilateral standard rTMS or bilateral TBS targeting the dorsolateral prefrontal cortex. The primary outcome was change in the Beck Scale for Suicide Ideation (SSI). The secondary outcome was remission of SI. Demographic, cognitive, and clinical characteristics that may moderate the effects of rTMS or TBS on SI were explored.

**Results:**

There was a statistically significant change in the total SSI score over time [*χ*^2^(7) = 136.018, *p* < 0.001], with no difference between the two treatment groups. Remission of SI was 55.8% in the standard rTMS group and 53.7% in the TBS group. In the standard rTMS group, there was no difference in remission of SI between males and females, whereas remission was higher in females in the TBS group (χ^2^(1) =6.87, *p* = 0.009). There was a significant correlation between time to remission of SI and RCI *z*-score for D-KEFS inhibition*/*switching [*r_s_* = −0.389, *p =* 0.012].

**Conclusions:**

Both bilateral rTMS and bilateral TBS were effective in reducing SI in LLD. There may be sex differences in response to TBS, with females having more favorable response in reducing SI. There may be an association between improvement in cognitive flexibility and inhibition and reduction of SI.

## Introduction

Several studies have established that repetitive transcranial magnetic stimulation (rTMS) is an effective and well-tolerated treatment for late-life depression (LLD) [1, 2]. Patients with LLD, particularly men, have a high risk of death by suicide [3]. There is evidence supporting the use of electroconvulsive therapy (ECT) or ketamine to treat suicidality; however, access to these treatment options is limited and both have possible adverse effects on cognition [4, 5]. Thus, there is a need for other effective and safe treatment options to address suicidal ideation (SI) in LLD.

A previous systemic review of 16 studies showed that rTMS is a safe and effective method for treating suicidal behavior [6]. It also found that the most effective form of treatment for suicidal behavior seems to be bilateral rTMS, particularly when combined with antidepressants [6]. A recent meta-analysis of 10 RCTs also reported that rTMS significantly reduced SI with an effect size (Hedge’s g) of −0.390 [7]. In another meta-analysis of 8 RCTs, SI scores were significantly lower in those who were treated with rTMS compared to sham control group [8]. However, the main limitation of the published systematic reviews and meta-analyses were heterogeneity in the stimulation parameters and variability in clinical measures used to report suicidal symptoms. Studies utilizing reliable and valid scales of suicidal symptoms are needed to further examine the effects of rTMS on suicidality.

Theta Burst Stimulation (TBS) is a newer, patterned form of rTMS that is modeled on endogenous brain oscillations [9]. TBS can achieve similar or more potent effects on synaptic facilitation in 1/10 time of the standard rTMS [10]. In a large multi-center clinical trial of adults (age 18–65 years) with MDD (the THREE-D trial), intermittent TBS (iTBS) of left DLPFC was shown to achieve a similar reduction in depressive symptoms compared to standard 10 Hz rTMS [11]. In a secondary analysis using the THREE-D trial data, our group found that both 10 Hz rTMS and iTBS were also similarly effective in reducing SI [12]. In a follow-up trial in patients with late-life depression, (the FOUR-D trial; ClinicalTrials.gov identifier: NCT02998580), our group found that bilateral sequential TBS achieved a non-inferior reduction in depression symptoms compared to standard bilateral rTMS [2]. However, the effects of TBS on SI in LLD have not been well characterized in the literature.

A previous study of mixed-age adults who had previously attempted suicide found that older age, female sex, and being free of sleep problems and major depressive episode were associated with higher odds of remission from SI [13]. Multiple studies have also shown that executive impairments, including decreased cognitive flexibility and inhibition, are associated with SI [14–17]. However, predictors of remission from suicidal ideation with rTMS in older adults have not yet been examined. The identification of factors that influence the effect of rTMS or TBS on SI may guide treatment decisions, such as the selection of specific rTMS protocols.

In this context, we examined the effects of standard bilateral rTMS and bilateral TBS on SI using data from the FOUR-D trial [2]. We hypothesized that both modalities would result in similar reduction of SI, as both have similar treatment effects on depression [2]. We also explored whether demographic, cognitive, and clinical characteristics may moderate the effects of rTMS or TBS on SI.

## Methods

This was a secondary analysis of data from the FOUR-D trial (ClinicalTrials.gov identifier: NCT02998580). Full details of the protocol have been reported previously [2]. In brief, FOUR-D study was a randomized non-inferiority clinical trial comparing the effectiveness of standard sequential bilateral rTMS and bilateral TBS in older adults (age 60 and older) with MDD. Specifically, the participants were outpatients with a moderately severe depressive episode (Montgomery-Åsberg Depression Rating Scale score 18 or higher), with nonresponse to 1 or more antidepressant trials of adequate dose and duration or intolerance of 2 or more antidepressants. Patients with SI were included unless they had active suicidal intent. The trial was conducted at an academic health center (Center for Addiction and Mental Health) in Toronto, Ontario, Canada. The study was approved by the research ethics board. All participants provided written informed consent to participate.

Participants were randomized to receive standard bilateral rTMS or bilateral TBS. Standard bilateral rTMS consisted of 1 Hz stimulation (120% resting motor threshold [RMT], 600 pulses over 10 minutes) to the right DLPFC, followed by 10 Hz stimulation (120% RMT, 3000 pulses: 4 seconds on, 26 seconds off over 37.5 minutes) to the left DLPFC. Sequential bilateral TBS consisted of continuous TBS (cTBS; 120% RMT, triplet burst pulses at 50 Hz, repeated at 5 Hz for 600 pulses over 40 seconds) to right DLPFC, followed by intermittent TBS (iTBS; triplet burst pulses at 50 Hz, repeated at 5 Hz, 2 seconds on, 8 seconds off, for 600 pulses over 3 minutes 9 seconds) to left DLPFC. Participants initially received 20 daily sessions over 4 weeks. They were offered an additional 10 daily sessions over 2 additional weeks if they did not achieve remission after 20 sessions. Participants who missed scheduled treatment days received the entire 20 to 30 treatments over an extended period. Those who missed more than 3 consecutive treatments were withdrawn.

The Beck Scale for Suicide Ideation (SSI) [18] was used to assess SI at baseline, weekly during treatment, at completion of treatment, and during longitudinal follow-up assessments (1, 4, and 12-weeks after treatment completion). The Montgomery-Åsberg Depression Rating Scale (MADRS) [19] was used to assess depressive symptoms at the same intervals and was the primary clinical outcome measure of the clinical trial.

A cognitive battery was administered at baseline, end of treatment, and 12 weeks post-treatment. Global cognition was assessed with the Montreal Cognitive Assessment (MoCA) [20]. Executive function was assessed with the National Institutes of Health (NIH) Toolbox Flanker Inhibitory Control and Attention Test [21] and Delis-Kaplan Executive Function System (D-KEFS) Color-Word Interference (CWI) test [22]. To expand further, there were four conditions of the D-KEFS CWI test, which measure the following: information processing speed (Conditions 1 and 2), inhibitory control (Condition 3), and inhibitory control*/*cognitive flexibility (Condition 4) [22]. Raw scores for cognitive measures were converted into age-corrected standard/scaled scores. Practice-adjusted reliable change index (RCI) z-score changes (from baseline to end of treatment) were calculated for the Flanker test and 4 conditions of the D-KEFS CWI, as described previously [23].

For the analyses, only participants who had SI (i.e., non-zero score on the baseline SSI) were included. Analyses were completed using IBM SPSS version 29 (Armonk, NY: IBM Corp). Intention-to-treat principle was followed, and missing data were imputed using the last observation carried forward method. Due to the non-normality of data, which could not be corrected using transformations, non-parametric tests were used for the analyses.

The primary outcome of this study was the change in SI over time, as measured by the change in SSI total score. Friedman test (non-parametric analog of one-way repeated measures ANOVA) was used with time as the independent variable and SSI total score as the dependent variable. Mann–Whitney *U* test was used to compare the change in SSI score from baseline to end of treatment between the two treatment groups (standard rTMS vs. TBS).

The secondary outcome of this study was remission of SI, defined as a score of zero on SSI at treatment completion. Chi-square test was used to compare the rates of remission between the two treatment groups. Mann–Whitney *U* test was used to compare time to remission (in weeks) between the two groups. We also examined the durability of response by comparing the recurrence of suicidal ideation (in those who had remission of SI) between the treatment groups during the 12-week follow-up period using chi-square test.

As exploratory analyses, we examined the relationship between demographic, cognitive, and clinical characteristics, and the effects of rTMS or TBS on SI. Spearman”s rank-order correlations were run to determine the relationship between the changes in SSI total scores (from baseline to end of treatment) and changes in modified MADRS scores (which excluded the suicide item on the MADRS), baseline MADRS scores, and age. Chi-square test was used to explore whether two treatment groups differed on the proportion of female and male participants who had remission of SI. Fisher’s exact test was also performed to assess for differences between treatment groups in the proportion of participants who had an increase in SSI score.

To examine the association between executive function/global cognition and change in SI, Spearman”s rank-order correlations were run between change in SSI score and cognitive measures (at baseline and change from baseline to end of treatment). Baseline cognitive measures that were included in the analyses were the MoCA, Flanker Inhibitory Control and Attention Test, and D-KEFS CWI inhibition/switching (condition 4). Practice-adjusted RCI z-scores for the Flanker Test and D-KEFS inhibition/switching were included. The same analyses were used to study the association between time to remission of SI and cognitive measures.

## Results

Of 172 participants enrolled in the FOUR-D trial (84 in standard rTMS group and 85 in TBS group), 84 had an SSI score of 1 or above at baseline and were included in the analysis. There were 43 participants in the standard rTMS group and 41 in the TBS group. Participant characteristics are shown in Table 1. Of the 88 participants who had an SSI score of 0 at baseline, 5/44 (11.4%) in the rTMS group and 7/55 (15.9%) had increased SI scores. There was no statistically significant difference between groups in the rate of increased SI [*χ*^2^(1) = 0.386, *p* = 0.534].

**Table 1.** Demographic, clinical factors, and changes in suicidality in older depressed patients treated with standard bilateral rTMS or bilateral TBS

		Standard bilateral rTMS*n* = 43	Bilateral TBS*n* = 41
Age, mean (range, SD)		67.3 (60–89, 7.2)	66.6 (60–81, 5.8)
Self-reported Sex, *n* (%)	Female	24 (55.8)	24 (58.5)
	Male	19 (44.2)	17 (41.5)
Race, *n* (%)	White	38 (88.4)	39 (95.1)
	Black	0 (0)	0 (0)
	Asian	4 (9.3)	1 (2.4)
	Other	0 (0)	1 (2.4)
	Prefer not to answer	1 (2.3)	0 (0)
Years of education, mean (SD)		15.5 (2.4)	15.3 (2.9)
Age of onset of MDD, mean (SD)		31.5 (20.8)	32.2 (18.9)
Major depressive episode duration in months, mean (SD)		46.8 (59.1)	49.5 (59.9)
Baseline MADRS total score, mean (SD)		27.1 (4.3)	27.3 (5.5)
Baseline SSI total score, mean (SD)		5.2 (6.3)	4.4 (5.2)

Abbreviations: MADRS, Montgomery-Åsberg Depression Rating Scale; SSI, Beck Scale for Suicide Ideation; rTMS, repetitive transcranial magnetic stimulation; TBS, theta burst stimulation.

**Figure 1.**
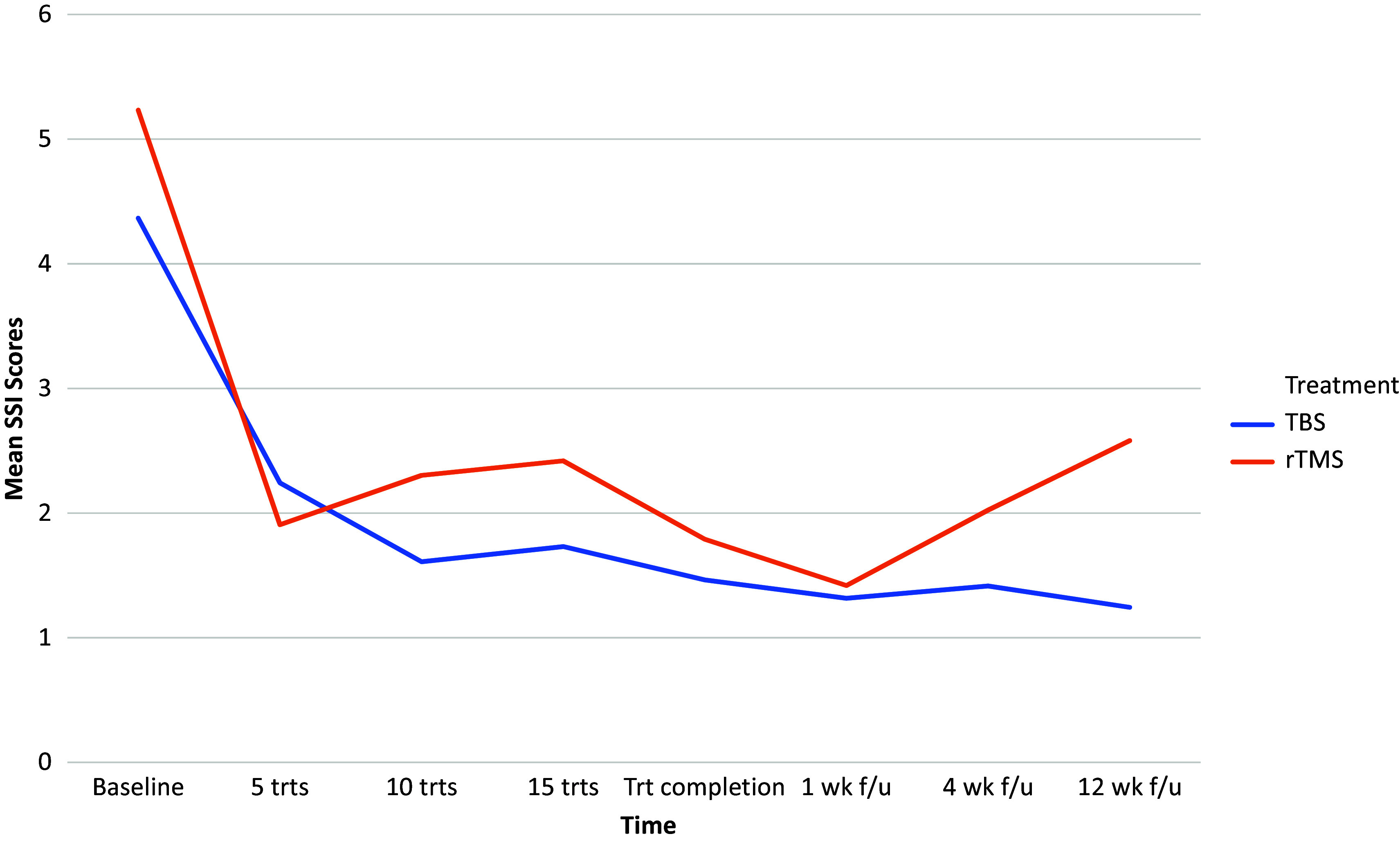
Mean SSI scores over time by treatment groups.

At the end of treatment, the mean (SD) reduction in the SSI total score was −3.44 (5.59) in the standard bilateral rTMS group and − 2.90 (4.89) in the bilateral TBS group. The mean change in SSI scores by treatment group over time is shown in Figure 1.

There was a statistically significant change in the total SSI score over time [χ^2^(7) = 136.018, *p* < 0.001]. There was no statistical difference between the two treatment groups (standard bilateral rTMS vs. bilateral TBS) in the change in SSI scores from baseline to end of treatment (*U* = 871, *p =* 0.924).

The overall rate of remission of SI was 46/84 (54.8%). There was remission of SI in 24/43 (55.8%) participants in the standard rTMS group and 22/41 (53.7%) participants in the TBS group. There was no statistically significant difference in the rate of remission between the treatment groups [χ^2^(1) = 0.04, *p* = 0.843]. The mean (SE) time to remission was 3.5 (0.4) weeks in the standard rTMS group and 2.5 (0.4) weeks in the TBS group. There was no statistically significant difference between groups in time to remission (*U* = 345.5, *p* = 0.064). Of those who had remission of SI, there was recurrence of SI in 9/24 participants in the standard rTMS group and 9/22 in the TBS group. This difference was not statistically significant [χ^2^(1) = 0.056, *p* = 0.813].

There were no statistically significant correlations between the changes in SSI total scores and changes in modified MADRS scores [*r_s_* = 0.180, *p =* 0.106], baseline MADRS scores [*r_s_* = −00.010, *p =* 0.926], or age [*r_s_* = −0.004, *p =* 0.969].

In the overall sample, 15/46 (41.6%) males achieved remission of SI, compared to 31/48 (64.6%) females (χ^2^(1) = 4.36, *p* = 0.037). In the standard rTMS group, 10/19 (52.6%) males achieved remission of SI compared to 14/24 (58.3%) females (χ^2^(1) = 0.14, *p* = 0.71). In the TBS group, however, 5/17 (29.4%) males achieved remission of SI compared to 17/24 (70.8%) females (χ^2^(1) = 6.87, *p* = 0.009).

There were no statistically significant correlations between the changes in SSI total scores and baseline MoCA scores, baseline age-corrected standard score for the Flanker task, baseline age-corrected scaled score for D-KEFS inhibition*/*switching, RCI z-score for the Flanker task, or RCI z-score for D-KEFS inhibition*/*switching. There were no statistically significant correlations between time to remission and baseline cognitive measures (MoCA, Flanker, D-KEFS inhibition/switching) and RCI *z*-score for the Flanker task. There was a significant correlation between time to remission of SI and RCI *z*-score for D-KEFS inhibition*/*switching [*r_s_* = −0.389, *p =* 0.012].

There was an increase in SSI score from baseline to end of treatment in 4/43 (9.3%) participants in the standard rTMS group and 4/41 (9.8%) participants in the bilateral TBS group, with no significant difference between groups (*p* = 1.00). No participants had to discontinue due to the development of suicidal intent.

## Discussion

In this secondary analysis, both standard bilateral rTMS and bilateral TBS resulted in a clinically meaningful reduction in SI in LLD, with no significant differences between the two treatment groups. There were no differences between these groups in the rates of remission of SI, time to remission of SI, or rates of recurrence of SI during the 12-week follow-up. However, a significantly higher proportion of females than males experienced remission of SI in the TBS group. This sex difference was not present in the standard rTMS group. These findings suggest that standard rTMS and TBS are similarly effective in reducing SI, though TBS may exert a differential effect in females compared to males. In addition, the time to remission of SI was negatively correlated with improvement in cognitive flexibility and inhibition.

Our finding that both standard rTMS and TBS are similarly effective in reducing SI in a large sample of depressed older adults is consistent with previously reported findings from a study of mixed-aged participants with MDD who received rTMS or iTBS [12]. In that study, which used the data from the THREE-D trial, the rates of remission of suicidal ideation were 40.4% with 10 Hz rTMS and 49.1% with iTBS of left DLPFC. In another study evaluating the effects of rTMS, SI resolved in 40.4, 26.8, and 18.8% of participants randomized to bilateral, left unilateral, and sham rTMS, respectively [24]. It was found that the difference between bilateral and sham were significant, unlike the difference between left unilateral and sham. The overall rate of remission of SI in our study was 54.8% (55.8% in standard rTMS and 53.7% in TBS), which is numerically higher than the previously reported rates of remission in mixed-age adults. The higher rates of remission of SI seen in this study suggest that older adults can also achieve clinically meaningful improvement in an important symptom domain with rTMS/TBS compared to younger adults.

In our study, the typical time to remission of SI was 2–3 weeks. There is growing interest in rapid treatment options for suicidality. In a cross-over RCT in younger patients with MDD (*N* = 81), accelerated iTBS (i.e., multiple treatment sessions in a day) with 40 sessions of stimulation over 2 weeks resulted in a significant reduction in SI scores [25]. In an open-label study in younger patients with MDD (*N* = 31), accelerated rTMS was associated with a suicidality remission rate of 77.42% within 5 days of treatment [26]. Future studies are needed to assess whether accelerated rTMS/TBS can also reduce suicidality in older adults with MDD in days rather than in weeks.

In both treatment groups, there was a recurrence of SI during the 12-week follow-up period in approximately 40% of the participants who initially achieved remission. This recurrence of symptoms suggests that future studies are needed to inform strategies to maintain the remission of SI following an acute course of treatment.

In our analysis, the reduction in SI (i.e., change in the total SSI score) was weakly and not significantly correlated with a decrease in severity of depression (i.e., change in the total modified MADRS score). In a previous retrospective analysis of veterans who received rTMS for depression, it was found that depressive symptom change did not always account for improvements in suicidal ideation [27]. In another study, there was a correlation between change in SI and change in depression severity; however, this correlation was modest (change in depression severity accounting for approximately 15% of the change in SI). Further, there was no difference in the change of 17-item Hamilton Depression Rating Scale score between suicide remitters and non-remitters [24]. Our findings suggest that reduction in suicidal symptoms is independent of treatment of depression, and that there may be discrete circuitry that needs to be engaged to optimally treat depression and suicidality. A previous analysis of an open-label trial of left DLPFC rTMS found a correlation between reduction in suicidality and functional connectivity between the dorsal striatum and frontopolar cortex, a circuit linked to impaired decision-making [28]. Very few analyses have explored the reduction of suicidality with rTMS applied to regions other than the left DLPFC. Future studies should consider investigating the effects of rTMS stimulation of various cortical areas, including the right DLPFC, on suicidality.

Our results also suggest that there may be a higher rate of remission of SI in older females than males with TBS, but not standard rTMS. Except for a few animal studies [29] and some indirect evidence [30], sex differences in rTMS studies and their potential impact on suicidality have not been studied. Given the paucity of evidence, prospective trials are needed to confirm sex differences in the effect of TBS on suicidal symptoms and to explore the underlying biological mechanisms.

There was a significant correlation between time to remission and RCI z-score for D-KEFS inhibition*/*switching task. Improvement in cognitive flexibility and inhibition from baseline to end of treatment was associated with shorter time to remission of SI. Multiple studies have found that there is an association between suicidal ideation and executive function impairments [14–16]. One cross-sectional study of 40 middle-aged and older adults with depression, which used the D-KEFS trail making test and color-word interference test, found that those with passive SI performed significantly worse on cognitive flexibility and inhibitory ability compared to those without passive SI [17]. To the best of our knowledge, there have been no studies to date that have looked at the association between executive functioning and remission of SI. However, a previous large study of over 400 older adults who received venlafaxine XR monotherapy for major depression found that those with “high and persistent SI” had worse scores on CWI inhibition*/*switching subtest of D-KEFS compared to those with “rapidly decreasing SI” [31]. This is in line with our finding that improved performance on the D-KEFS CWI inhibition/switching was negatively correlated with time to remission of SI.

Our analysis has several strengths and limitations. Strengths include that the data were from a randomized trial using a specific and validated suicidal measure, the SSI. One of the major limitations of this study is the lack of a control group, as all participants were treated with active treatment with either standard rTMS or TBS. Another limitation of this study is that participants with active suicidal intent were excluded, and the baseline SSI scores were low (5.2 in the standard rTMS group and 4.4 in the TBS group). Future studies with those with more severe suicidal symptoms are needed to explore if rTMS can be used to treat more severe suicidality, similar to ECT and ketamine. In a pooled analysis of two RCTs in mixed-age adults with treatment-resistant MDD (*N* = 153), low-dose ketamine infusion and unilateral iTBS were similarly effective in treating suicidal symptoms, and more effective than saline infusion, unilateral 10 Hz rTMS, or sham rTMS [32]. In one RCT (*N* = 73), bilateral ECT was superior to unilateral rTMS in reducing suicidal scores [33]. As such, future studies are needed to compare the effectiveness of emerging interventional psychiatry approaches in targeting suicidal symptoms in older depressed patients.

Another limitation is that suicidality was not the primary aim of the original trial but an exploratory aim. Finally, the only targeted cortical area was the bilateral DLPFC; as discussed above, it is unclear whether there may be other cortical targets that may be more beneficial or more enduring for the treatment of suicidality.

In conclusion, our findings support that standard bilateral rTMS and bilateral TBS are both effective in reducing SI in older adults with MDD. It suggests that there may be possible sex differences in response to TBS, with female participants having more favorable response to TBS in reducing SI. Our findings also suggest that improvement in cognitive flexibility and inhibitory ability is associated with faster remission of SI with rTMS and TBS. As such, demographic, cognitive, and clinical characteristics, including detailed assessment of suicidal symptoms, should be taken into consideration when assessing the suitability of an older adult with MDD for treatment with rTMS and selecting treatment protocols.

## Supporting information

10.1192/j.eurpsy.2025.10049.sm001 Lee et al. supplementary material Lee et al. supplementary material

## Data Availability

The data that support the findings of this study are not openly available due to reasons of sensitivity. Data are located in controlled access data storage at the Centre for Addiction and Mental Health. Further inquiries can be directed to the corresponding author.

## Supplementary material

The supplementary material for this article can be found at http://doi.org/10.1192/j.eurpsy.2025.10049.
