# Supplementary material for: Clinical effects and correlates of standard rTMS and theta burst stimulation (TBS) on suicidal ideation in late-life depression
Source: Eur Psychiatry. 2025 Jun 27;68(1):e92. doi: 10.1192/j.eurpsy.2025.10049 (PMC12260718; doi:10.1192/j.eurpsy.2025.10049)
Supplement: Lee et al. supplementary material [file S0924933825100497sup001.docx]

**SUPPLEMENTARY MATERIAL**

*Inclusion criteria*: outpatient adults 60 years and older with major depressive disorder confirmed by the Mini-International Neuropsychiatric Interview, moderate depression severity based on MADRS score 18 or higher, nonresponse to 1 or more antidepressant trial of adequate dosage and duration using the Antidepressant Treatment History Form or intolerance of 2 or more antidepressants, no increase or initiation of psychotropic medication 4 weeks prior to screening, and normal pre-study bloodwork results

*Exclusion criteria*: substance misuse or dependence within the last 3 months, unstable physical illness, active suicidal intent, current psychotic symptoms, bipolar disorder, other psychiatric disorder causing greater impairment than major depressive disorder, dementia, Short Blessed Test total score of more than 10, electroconvulsive therapy or rTMS during the current episode, seizure disorder or lesion-related seizure, intracranial implant or metal in the cranium, implanted electronic device, and anticonvulsant use or benzodiazepine more than or equal to lorazepam 2 mg/d equivalents
